# Preduodenal portal vein: a case series of variable clinical presentations and surgical implications

**DOI:** 10.1186/s12887-025-06009-5

**Published:** 2025-09-09

**Authors:** Ahmed Arafa, Abdelhafeez Mohamed Abdelhafez, Ahmed S. Ragab, Abdelhalem Showkat Mohamed

**Affiliations:** 1 Pediatric Surgery Department, Faculty of Medicine, Cairo University, Cairo, Egypt; 2 Pediatric Surgery Department, Faculty of Medicine, Beni Suef University, Beni Suef, Egypt; 3 Pediatric Surgery Department, Faculty of Medicine, Port Said University, Port Said, Egypt; 4 Pediatric Surgery Department, Faculty of Medicine, Minia University, Minia, Egypt

**Keywords:** Preduodenal portal vein, Kasai, Duodenoduodenostomy

## Abstract

**Aim of the study:**

To present a case series of four pediatric patients with PDPV, each with a different clinical presentation and surgical management.

**Methods:**

We retrospectively reviewed four cases of PDPV managed at our institution. Two cases were associated with extrahepatic biliary atresia (EHBA) and discovered incidentally during surgery. The other two cases presented with duodenal obstruction but had differing etiologies and management approaches.

**Results:**

Two patients with EHBA underwent successful Kasai portoenterostomy; PDPV was discovered intraoperatively and required no intervention. One patient had duodenal obstruction due to PDPV compressing the duodenum and underwent laparoscopic duodenoduodenostomy. Another patient had duodenal obstruction due to malrotation; a Ladd’s procedure was performed. PDPV was noted intraoperatively but was not the obstructing factor.

**Conclusion:**

PDPV can present variably, ranging from an incidental finding to a causative factor in duodenal obstruction. Its recognition is crucial during abdominal surgery to avoid inadvertent injury and to tailor the surgical approach based on associated anomalies.

## Introduction

Preduodenal portal vein (PDPV) is a rare embryologic vascular anomaly characterized by an anomalous anterior course of the portal vein across the duodenum. It is frequently associated with other congenital abnormalities, including duodenal atresia, malrotation, biliary atresia, polysplenia, and heterotaxia syndrome [1]. PDPV may be discovered incidentally or present with obstructive symptoms, depending on its anatomical relationship to surrounding structures. Early recognition is vital for appropriate surgical management, particularly during procedures such as the Kasai operation or duodenal bypass surgery.

## Methods

### Case presentations

**Fig. 1:**
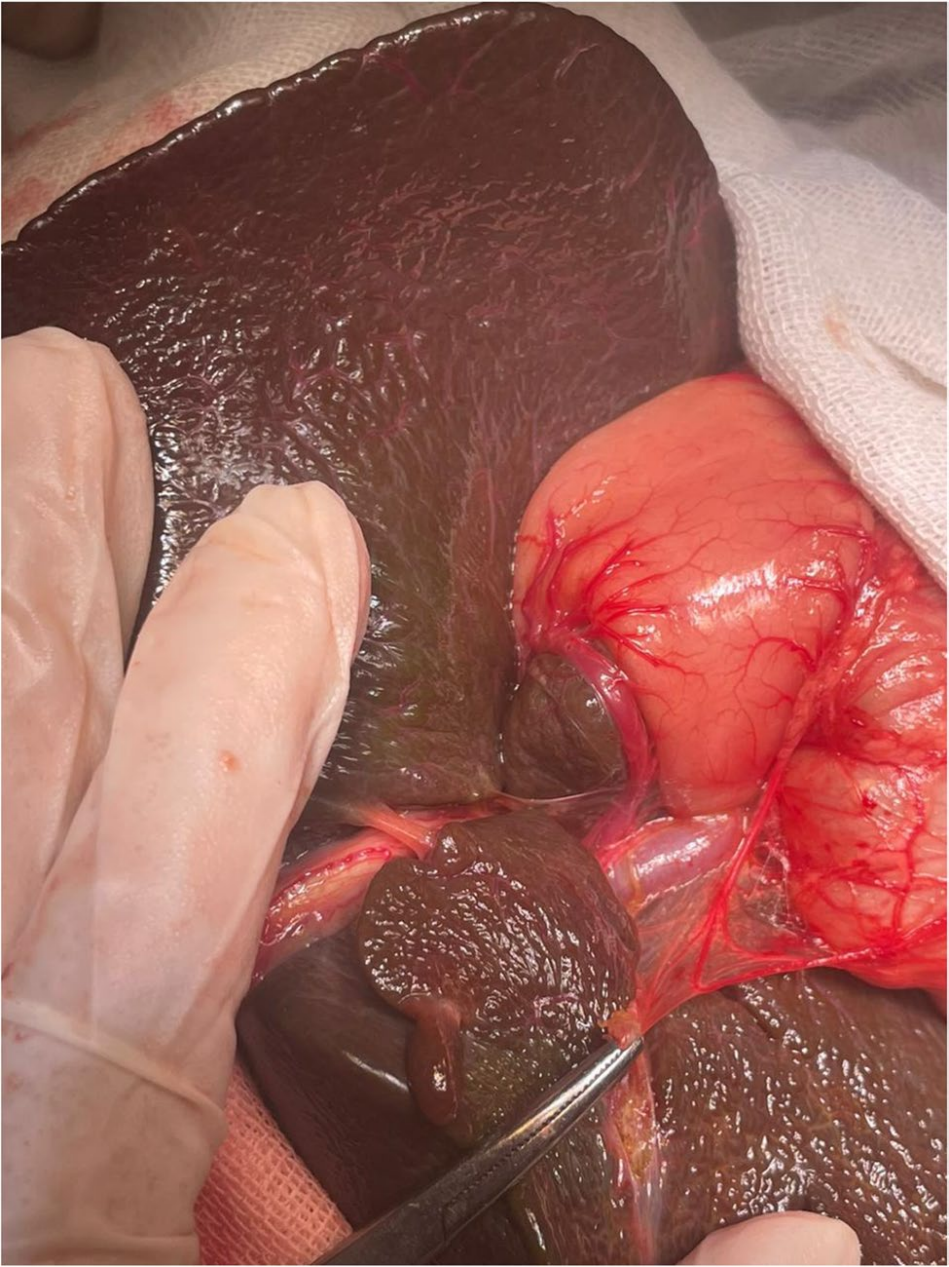
PDPV associated with EHBA

Cases 1 & 2: PDPV with Extrahepatic Biliary Atresia (Fig. 1)

 Both patients presented with neonatal jaundice and pale stools. Liver biopsy confirmed EHBA. During laparotomy for Kasai portoenterostomy, PDPV was discovered coursing anterior to the duodenum. The vein was not causing obstruction and was left intact. Both patients tolerated the procedure well with no complications related to PDPV.

Case 3: PDPV Causing Duodenal Obstruction

**Fig. 2:**
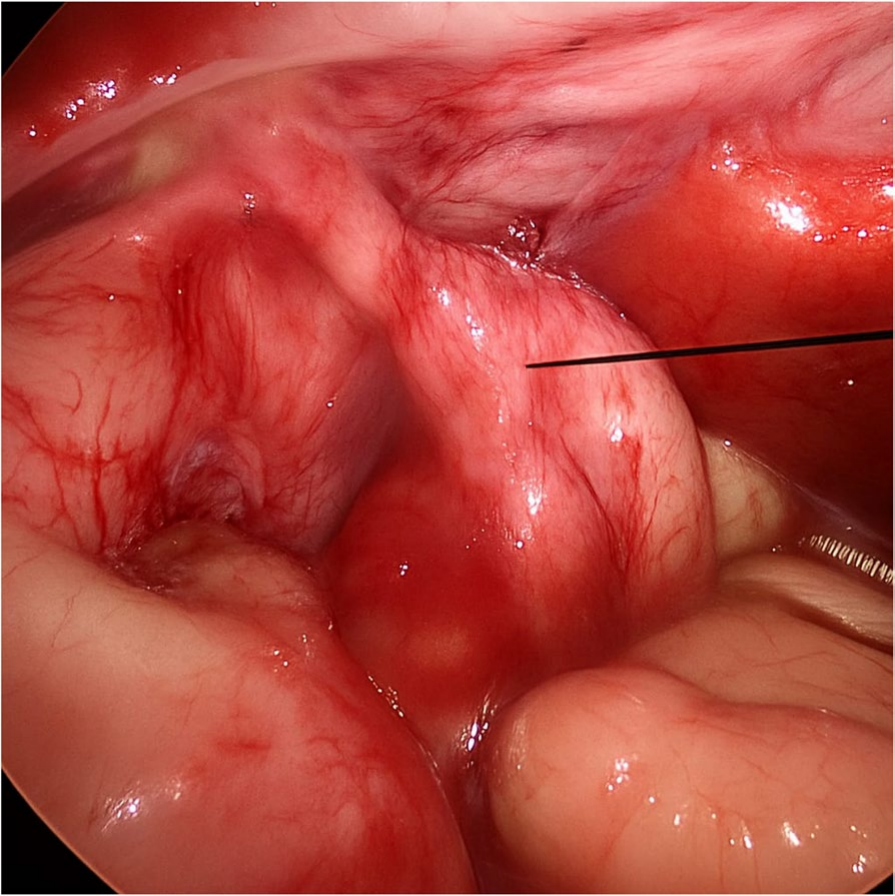
Arrow showing PDPV

**Fig. 3:**
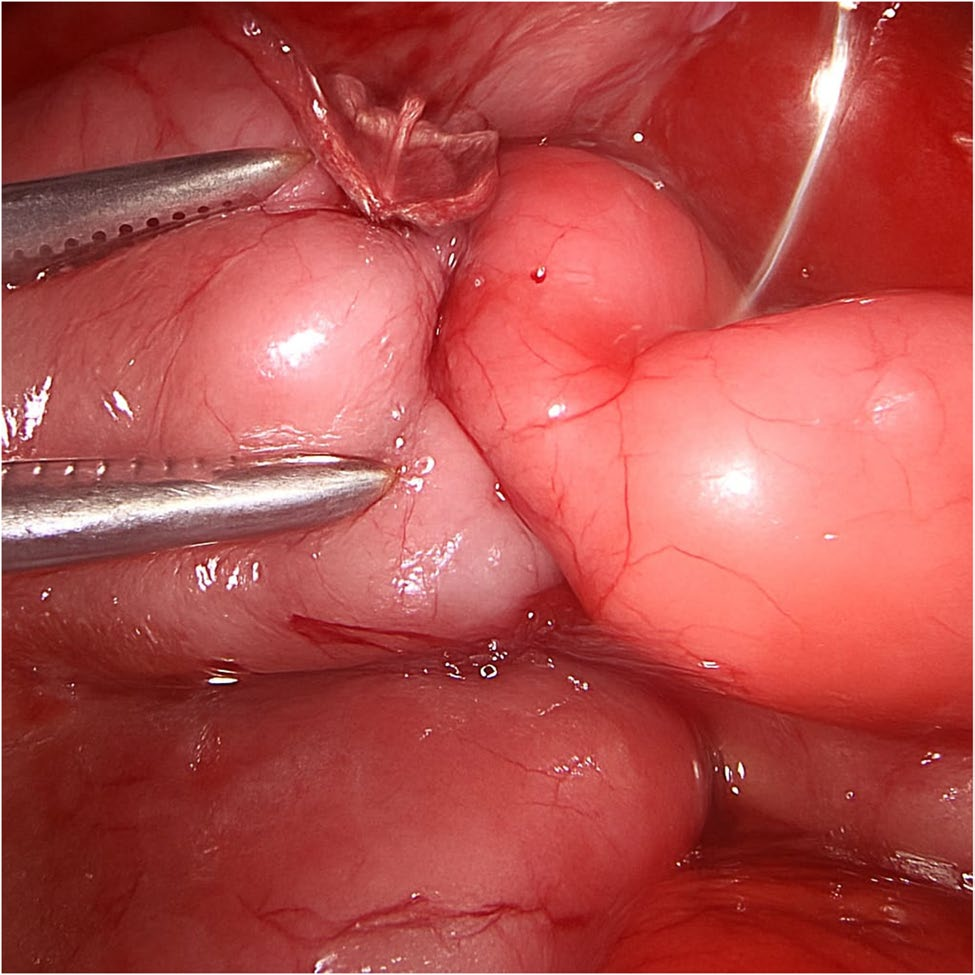
Laparoscopic duodenoduodenostomy

 A newborn presented with bilious vomiting. Imaging suggested duodenal obstruction. During diagnostic laparoscopy, a PDPV was found compressing the second part of the duodenum. A laparoscopic duodenoduodenostomy was performed anterior to the PDPV (Figs. 2, 3). The patient recovered well with resolution of symptoms.

**Fig. 4:**
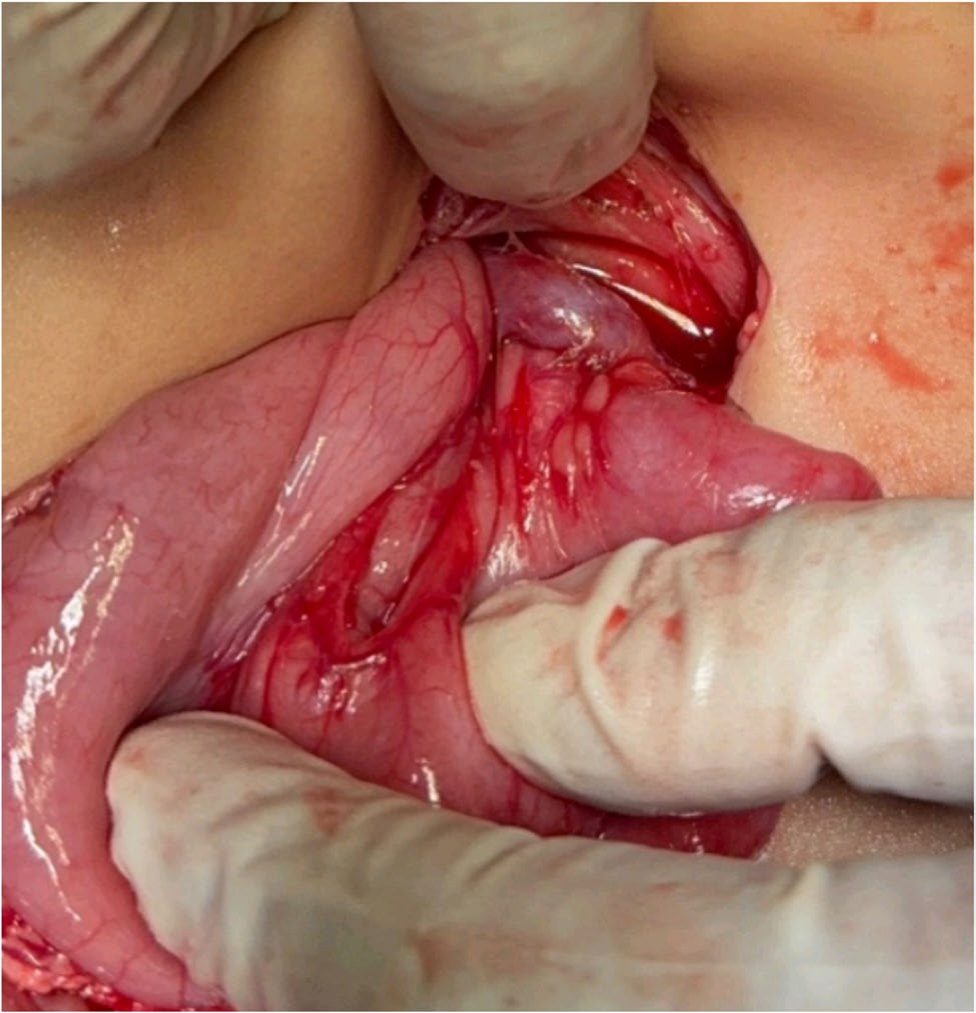
PDPV associated with malrotation

Case 4: Duodenal Obstruction Due to Malrotation with Incidental PDPV (Fig. 4)

 Another neonate presented with features of duodenal obstruction. Laparoscopic exploration revealed malrotation; the procedure was converted to open surgery. Ladd’s bands compressing the duodenum were found and divided. A PDPV was also noted but was not the cause of obstruction. Ladd’s procedure was completed successfully, and the PDPV was preserved. Table 1.

**Table 1:** Different presentations of PDPV

Case	Presentation	Diagnosis	Type of operation	Age at operation	Birth weight	Operative time	Complication	Hospital stay
1	Clay-colored stool, jaundice	EHBA	Kasai	2 months	5 kg	3 h	No	1 week
2	Clay-colored stool, jaundice	EHBA	Kasai	2 months	6 kg	3 h	No	1 week
3	Bilious vomiting	Duodenal obstruction due to PDPV	Duodenoduodenostomy	5 days	4 kg	2 h	No	4 days
4	Bilious vomiting	Duodenal obstruction due to malrotation	Ladd’s Procedure	6 days	3.5 kg	2 h	No	5 days

## Results

### Discussion

Preduodenal portal vein (PDPV) was first described by Knight in 1921 [1]. It is a rare anomaly, with only around 100 cases reported across different age groups; 29 cases have been documented in adults [2, 3, 8]. PDPV is characterized by anterior positioning of the portal vein relative to the duodenum, due to abnormal embryologic development—specifically, persistence of the ventral anastomosis of the vitelline veins and regression of the dorsal one.

Our case series highlights the variability in clinical presentations and surgical implications associated with PDPV. Ishizaki et al. [2] and Ooshima et al. [3] noted that approximately half of PDPV cases are asymptomatic and found incidentally, which aligns with our experience. In our series, two cases were incidentally discovered during surgery for EHBA.

The use of laparoscopic approaches in our cases mirrors current trends in neonatal surgery, with benefits including faster recovery and improved cosmetic outcomes.

The association between PDPV and EHBA, though uncommon, has been reported. Yamagiwa et al. [5] described a similar case during a Kasai operation, emphasizing the importance of careful dissection. In our two EHBA cases, PDPV was noted intraoperatively but did not interfere with portoenterostomy.

In Case 3, PDPV was the primary cause of duodenal obstruction. A laparoscopic duodenoduodenostomy was successfully performed, consistent with reports by Georgacopulo et al. [6]. Again, laparoscopy proved effective for both diagnosis and treatment.

In Case 4, PDPV was incidentally discovered during a Ladd’s procedure for malrotation. As seen in studies by Mordehai et al. [8] and Rusu et al. [9], PDPV in such settings may not be the obstructing element, and the primary surgical correction should address the actual cause of obstruction.

Yoon et al. [10] reported that preoperative imaging such as Doppler ultrasound or contrast-enhanced CT may help identify PDPV, although this is not always feasible in neonates. In our cases, PDPV was diagnosed intraoperatively, reinforcing the importance of surgical awareness and careful dissection.

Preoperative diagnosis remains a challenge. Routine imaging may miss PDPV, and contrast studies can be nonspecific. However, high suspicion and meticulous evaluation of imaging studies may lead to early recognition, especially when associated anomalies are present.

From a surgical perspective, early identification of PDPV is essential to avoid injury. Surgeons should remain vigilant for anomalous vascular courses, particularly in the context of duodenal obstruction or EHBA, to ensure safe and effective management.

## Conclusion

PDPV is a rare but clinically relevant anomaly, especially when associated with duodenal obstruction. Early intraoperative identification is vital for avoiding complications. Surgeons should maintain a high index of suspicion, particularly when other congenital anomalies are present.

## Data Availability

The datasets used and/or analyzed during the current study are available from the corresponding author on reasonable request.

## Abbreviations

EHBA: Extrahepatic biliary atresia
PDPV: Preduodenal portal vein
